# Development, design, and conceptual issues of project zero exposure: A program to protect young children from tobacco smoke exposure

**DOI:** 10.1186/1471-2458-11-508

**Published:** 2011-06-28

**Authors:** Laura J Rosen, Nurit Guttman, Melbourne F Hovell, Michal Ben Noach, Jonathan P Winickoff, Shosh Tchernokovski, Joseph K Rosenblum, Uri Rubenstein, Vered Seidmann, Constantine I Vardavas, Neil E Klepeis, David M Zucker

**Affiliations:** 1 Dept. of Health Promotion, School of Public Health, Sacker Faculty of Medicine, Tel Aviv University, POB 39040, Ramat Aviv 69978 Israel; 2 Dept. of Communications, Faculty of Social Sciences, Tel Aviv University, POB 39040, Ramat Aviv 69978 Israel; 3 Center for Behavioral Epidemiology and Community Health, Graduate School of Public Health, San Diego State University, San Diego, California, USA; 4 MGH Center for Child and Adolescent Health Policy, 50 Staniford St, Suite #901, Boston, MA 02114, USA; 5 Health Promotion Unit, Meuhedet, 124 Ibn Gavriol St, Tel Aviv, Israel 62038; 6 Medical Division, Meuhedet, 124 Ibn Gavriol St., Tel Aviv, Israel 62038; 7 Laniado Hospital, Netanya, Israel; 8 Department of Social Medicine, Faculty of Medicine, University of Crete, PO Box 2208, Heraklion, 71003, Crete, Greece; Center for Global Tobacco Control, Department of Society, Human Development and Health, Harvard School of Public Health, 677 Huntington Avenue, Boston, MA 02115, USA; 9 Education, Training, and Research, Inc., Scotts Valley, CA USA; and Department of Civil and Environmental Engineering, Stanford University, Stanford, CA, USA; 10 Dept. of Statistics, Hebrew University, Mt. Scopus, Jerusalem, Israel

**Keywords:** Tobacco smoke exposure (TSE), Secondhand smoke (SHS), tobacco control, children, health promotion, development of intervention, cluster randomized controlled trial

## Abstract

**Background:**

Tobacco smoke exposure (TSE) is a serious threat to child health. Roughly 40% of children worldwide are exposed to tobacco smoke, and the very young are often "captive smokers" in homes in which others smoke.

The goal of this research project is to develop and evaluate an intervention to reduce young child tobacco smoke exposure. The objective of this paper is to document our approach to building the intervention, to describe the planned intervention, and to explore the conceptual issues regarding the intervention and its evaluation.

**Methods/Design:**

This project is being developed using an iterative approach. We are currently in the middle of Stage 1. In this first stage, Intervention Development, we have already conducted a comprehensive search of the professional literature and internet resources, consulted with experts in the field, and conducted several Design Workshops. The planned intervention consists of parental group support therapy, a website to allow use of an "online/offline" approach, involvement of pediatricians, use of a video simulation game ("Dr. Cruz") to teach parents about child TSE, and personalized biochemical feedback on exposure levels. As part of this stage we will draw on a social marketing approach. We plan to use in-depth interviews and focus groups in order to identify barriers for behavior change, and to test the acceptability of program components.

In Stage II, we plan to pilot the planned intervention with 5-10 groups of 10 parents each.

In Stage III, we plan to implement and evaluate the intervention using a cluster randomized controlled trial with an estimated 540 participants.

**Discussion:**

The major challenges in this research are twofold: building an effective intervention and measuring the effects of the intervention. Creation of an effective intervention to protect children from TSE is a challenging but sorely needed public health endeavor. We hope that our approach will contribute to building a stronger evidence base for control of child exposure to tobacco smoke.

**Trial registration:**

ClinicalTrials.gov Identifier: NCT01335178

## Background

An estimated 40% of children worldwide are exposed to secondhand smoke (SHS) [1]. While exposure causes serious damage to both children and adults, infants and children are especially susceptible to SHS toxicity due to their size and developmental stage [2]. SHS exposure (SHSe) causes sudden infant death syndrome, reduced birthweight, and ottitis media, asthma, pneumonia, and impaired lung function in children [2]. Detrimental health effects of exposure to SHS have been shown to persist into adulthood, and children with parents that smoke are known to be at a greater risk for tobacco use themselves [3,4]. According to the World Health Organization, medical costs of children due to SHS have been estimated at $703-$897 million in the US, $239.5 million in Canada, and $267 million in Britain [5]. Because of its potential impact, reduction of children's SHS exposure is on the agenda of major health organizations, including the World Health Organization [1,6] and US Healthy People 2020 [7].

### Prior approaches to reducing SHS exposure among the very young

Largely due to the difficulties of legislating bans in the private domain [8], little legal action has been taken to prevent the exposure of young children to SHS in the home. The use of voluntary smoking bans in the homes and cars of families with children is one possible approach. Some evidence of public support for such am approach exists in the US and elsewhere. In the summer of 2001, for example, 74% of US households had indoor smoking bans, with 84% reporting smoking bans in the presence of children [9]; this is corroborated by another study showing strict smoking bans in just over three quarters of US households [10]. Support has been found not only for voluntary smoking restrictions, but also for those mandated by law. In Canada in 1996, nearly 40% of residents supported legal restrictions on home SHS child exposure [8]. The first ban on smoking with a child in the car, enacted several years ago in Bangor, Maine [11], was followed by bans in other places, including Australia [12].

Some researchers have attempted to develop intervention programs to reduce SHSe among children, particularly in the home. These interventions have had limited success in achieving their stated goals. In a 2006 Cochrane Collaboration review of 36 trials, only 11 showed statistically significant reductions in child exposure to SHS. The review states: "Although several interventions, including parental education and counselling programmes, have been used to try to reduce children's tobacco smoke exposure, their effectiveness has not been clearly demonstrated." [4]. The US Task Force on Community Preventive Services [13], in its review of community education to reduce exposure to SHS in the home, found "insufficient evidence to determine effectiveness ... because of the small number of available studies and limitations in their design and execution."

Techniques such as intensive behavioral counseling [5], motivational interviewing with children from low-income households [14], and a brief intervention with smoking mothers of newborns [15] have all shown some success at reducing exposure to SHS or reducing parental smoking. The potential role of pediatricians has also been highlighted [16], particularly because of their responsibilities in some countries to report suspected physical harm or neglect [17]. Some interventions in the pediatric setting have shown benefit, particularly for asthmatic children [18]. Other approaches have been found to be feasible, such as the STOP program for counseling parents in the hospital environment [19], or acceptable, such as physician advice for protecting children from SHS in the pediatric setting [20,21].

### Challenges related to parental perceptions and practices

Several important challenges have been identified in the literature on preventing children's exposure to SHS, in particular those associated with parental beliefs and practices regarding the impact of their own smoking on their children, or the effectiveness of the prevention measures which they employ. A central challenge is parents' denial of the detrimental effect of SHS caused by their own smoking. For example, in one study, some smoking mothers of young children, rather than admit their smoking could potentially affect their children, preferred to blame health-related issues on other factors, such as genetics or environmental pollution [22]. Another challenge is that even when parents do recognize the potential hazard of exposure to SHS, a large percentage (over 80% in one study [23]) rely on simplistic "harm-reduction" strategies which they believe are effective, such as opening a window. However, research shows such strategies to be ineffective [24]. These two types of misconceptions pose an important challenge in developing effective interventions: in addition to providing the parents with resources to adopt and maintain smoke-free environments for children, parental misconceptions regarding harm reduction must be addressed. For this purpose, a social marketing approach, which draws on behavior-change theories [25,26] will be used to augment findings from the comprehensive literature review, by systematically identifying parents' barriers to the adoption of recommended SHS-reduction behaviors [27]. In addition, it will use a formative evaluation strategy [28] to enable the development of appropriate theory-based means to address the challenges, which will avoid stigmatizing parents or making them feel guilty [29-31]. These behavior-change approaches include social cognitive theory [32], risk communication, the influence of social norms [33], and the use of various media channels (which currently include the internet and social network channels) to reach and attract the attention of the intended parent population, and to provide pertinent and persuasive information on the issue. Further, new media channels can play an important role in providing actual support and enhanced self-efficacy, which are considered to be important factors in adopting health-promoting behavior changes. Social marketing approaches also emphasize the importance of identifying subgroups (segments) within the intended population that may hold particular beliefs, have particular needs, or can be supported through particular means. This segmentation can be done on the basis of various factors, including particular "stage" of readiness to adopt the recommended behavior [34]. Social marketing strategies have been successfully applied in the area of tobacco use prevention and cessation, and it has been recommended that they be applied in the development of current smoking prevention approaches [4,35,36].

### Third hand smoke exposure

The concept of third-hand smoke (THS), defined as "residual tobacco smoke contamination that remains after the cigarette is extinguished," has recently surfaced as a closely related health issue. The term "tobacco smoke exposure" (TSE), is used to include exposure to both secondhand smoke and thirdhand smoke, and will be used in the remainder of this protocol [10].

### Primary hypothesis of the current study

The primary hypothesis of the current study is that a parent-oriented theory-based intervention can reduce tobacco smoke exposure (TSE) of young children, and be evaluated in a valid manner.

### Specific aims

The aims of the present research are to:

1. Develop a theory-based intervention based on a social marketing approach to reduce TSE among young children

2. Evaluate the effects of the intervention on TSE, as measured by biochemical measures and parental report

3. Evaluate the effects of the intervention on secondary endpoints: child health outcomes and parental cessation

4. Explore the relationship between TSE and child health

5. Explore the relationship between TSE as reported by parents and as measured by biochemical means.

## Methods/Design

### Project Phases

The development and evaluation of the intervention, detailed in Table 1, follows the model for the development of lifestyle interventions presented previously by Rosen et al. [37] The model specifies that the program be developed in phases. In Phase I, the intervention is constructed and tested with a small number of individuals, with an emphasis on acceptability and feasibility, using a qualitative approach to evaluation. The intervention is tested in Phase II in a real field setting, with a limited number of individuals, often iteratively, using a before-and-after design. In Phase III, the effectiveness of the intervention is tested, using a randomized design. In Phase IV, large-scale implementation and penetration are assessed in other populations. This project addresses Phases I-III.

**Table 1:** Phased, evaluated development plan for intervention to reduce child exposure to tobacco smoke

*Phase*	*Activity*
**I. Program development**	

	Systematic review of the professional literature (with meta-analysis)

	Comprehensive search for intervention materials available online,

	Consultation with experts

	Creation of initial version of website

	Development of research instruments (questionnaires and Interview Guides

	Focus groups and in-depth interviews with parents about attitudes and practices regarding child tobacco smoke exposure Qualitative evaluation

	Focus groups with parents on measurement of exposure Qualitative evaluation

	Design workshops to get feedback from parents on all elements of program. Qualitative evaluation

**II. Pilot**	Pilot all elements of intervention and research materials on 5-10 groups of 10 parents each. Qualitative and quantitative evaluation

**III. Cluster randomized RCT**	Randomize 18 clinics to early or late intervention, with 30 participants per clinic. Quantitative evaluation of primary and secondary endpoints, qualitative and quantitative process evaluation.

#### Phase I. Intervention Development

The following activities have been performed during Phase I: (1) a broad literature review, which encompassed reports of previous intervention trials, information provided by state and local governments for parents and professionals interested in protecting children from tobacco smoke exposure, and information about parental attitudes towards smoking around children; (2) consultations with leading international experts in the field, (3) discussions with local health, communications, and parenting professionals, and (4) two early design workshops on the topic of website development. Based on the information and experience obtained through these activities, we developed an initial intervention plan. The plan will be refined on the basis of formative research, consisting of personal and group interviews with parents and health professionals. The aim of these interviews is to identify beliefs and knowledge regarding smoking around children, perceived social norms regarding parental smoking around children, reliable information sources regarding smoking hazards and smoking cessation, channels of influence and support, and barriers obstacles to the adoption of smoking bans in the home and car.

Focus groups (group interviews) and in-depth personal interviews of parents will be conducted to elicit their perceptions of risk and exposure, identify obstacles to implementing smoke-free home and car policies, and develop ideas for fully protecting their children from the effects of TSE. The emphasis will be on the perspective and needs of the target population, including what parents understand about the damage caused by TSE, why parents continue to smoke around their children despite having some knowledge of the potential hazard of child TSE, and what resources they need to overcome the barriers that prevent them from adopting practices that can help protect their children from TSE. Phase I will also include preliminary testing of all program components.

#### Phase II: Pilot of developed intervention

In Phase II, we plan to conduct a pilot study to fully test all elements of the planned intervention program, as well as all evaluation tools. Because the planned intervention will be conducted with groups of parents, and not with individuals, we adapt the formulation of Phase II to accommodate work with groups. The original formulation of Phase II called for recruiting participants and then offering them the developed intervention. Instead, we plan to implement the intervention in groups of 10 families each. We plan to work with the first group of 10 parents, modify the program according to initial results, and then run the second group. This iterative approach to improving the program will continue for 5-10 sessions, until we have a feasible, well-accepted program with at least some evidence of decrease in exposure levels. This continued refinement of the program should allow us to fully and immediately incorporate findings from early group sessions, so that all modifications are tested prior to the Phase III trial. The disadvantage of this approach is that our estimate of intervention effect will be based on fewer participants.

#### Phase III. Cluster randomized controlled trial

**Figure 1:**
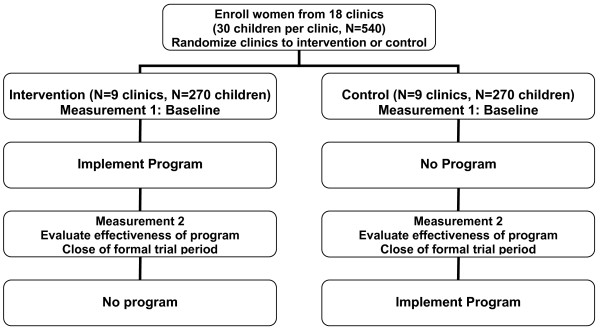
**Flowchart of Study Design**.

The study design for the Phase III trial is diagrammed in Figure 1. In brief, it is a cluster randomized design, with randomization at the level of the clinic, and delayed project implementation in the control group. Thus, the intervention group will receive the intervention soon after entry to the study, while the control group will receive the intervention at the close of the study, some six months later. This design, which was used previously in a trial of a preschool handwashing intervention by several of the investigators in the current research [38,39], enhanced the acceptability of the trial to potential participants, and overcame ethical objections to withholding possibly beneficial intervention from control group members [40].

### Population

The study will be conducted in Israel, where 20.9% of the adult population smokes [41]. While there are no nationally representative data on young child exposure to SHS, one study conducted in Haifa showed that an estimated 46% of infants less than one year old resided in homes with one or more smokers [42]. Forty percent of school-aged children in seventh through twelfth grade are exposed to SHS in their homes [43].

All Israeli citizens are covered by the National Health Insurance Law through one of four Health Maintenance Organizations (HMOs). Participants for in-depth interviews, focus groups, and the Pilot Study will be recruited through three clinics owned by Meuhedet, an HMO which serves a million individuals. The clinics are located in three different cities in Israel, all of which are located in the center of the country. Each of these clinics will give us a unique perspective on trying to control secondhand smoke exposure in different populations.

City X has a middle-income heterogeneous population, City Y has a primarily Ultra-Orthodox population, and City Z has a mixed-income heterogeneous population which includes a high percentage of immigrants from the former Soviet Union. Men from the former Soviet Union smoke more frequently than native-born Jewish Israelis [44]. The Ultra-Orthodox males have rates of smoking which are similar to other Jewish males, while the Ultra-Orthodox females have very low smoking rates. Consequently, in the City Y clinic, the emphasis will be on controlling exposure of children in a population where many fathers smoke but few mothers do, while in the other areas, the focus will be on households where either parent smokes or both parents smoke.

Potential participants coming for a visit to their Meuhedet clinic will be find posters and flyers advertising for participants in the project. In addition, they will be approached by the clinic staff (physicians, nurses, health promoters, or secretaries) and asked to agree to be contacted by project personnel for possible participation in the study. The eligibility criteria are as follows:

1. The child is a Meuhedet member, cared for in one of the participating clinics.

2. At least one parent is a current smoker.

3. The child is of the appropriate age (< 3 years of age).

4. The child has sufficient hair growth for hair samples.

5. The parents give consent for participation in the research.

We will attempt to contact all candidates, inform them about the study, and invite them to join. Participating parents will sign Informed Consent Forms.

### Intervention

#### General Focus

Previous work has focused on one of two primary goals: convincing parents to quit smoking or convincing them to reduce the exposure of their children [6]. Parental cessation is the ideal, but poses several significant problems regarding reducing child TSE: (1) Many parents are not interested in quitting. (2) Among parents who try to quit, many fail entirely and others relapse. (3) Even if one parent quits, another parent or household resident may continue to smoke, still leaving the child exposed, and (4) even if both parents quit, the child may still be exposed by others inside or outside the home.

These factors suggest that a focus on reducing exposure has important advantages. It would allow recruitment of a broader segment of parents, because parents who are not willing to consider quitting might also be interested in joining. In addition, the program would address elimination of all exposure to SHS, from any source. Further, it may help parents consider cessation, when they see they can be provided with a supportive environment that offers them the means to reduce their smoking or quit all together.

Drawing on these considerations, we have chosen a combined approach. Our main focus will be on reducing child TSE, but we will also include elements that will provide support for parental smoking cessation. Specifically, cessation will be encouraged and facilitated through referrals to existing cessation services within the healthcare system. It should be noted that group therapy for smoking cessation, along with cessation medications for those participating in group therapy, are heavily subsidized for all Israeli citizens.

#### Intervention Components

The main components of the planned intervention are as follows:

1. Group support sessions to help parents create and maintain smoke-free homes, and protect their children from exposure, run by experts in parenting skills

2. A project website which will be used in conjunction with the group support sessions. The parents will have access to information provided by the Project and will be able to engage in online discussions with project personnel and each other. This will create an "online/offline" approach to working with the parents.

3. Providing results of biochemical measures of child exposure as a means to illustrate to parents the extent of their child's TSE.

4. A video simulation game ("Dr. Cruz") developed to inform parents about exposure levels and risks of exposure while smoking in a car with a child present [45].

5. Informing participants' physicians about the project and providing them with information about the dangers of TSE to child health, including CEASE (Clinical Effort about Secondhand Smoke Exposure) materials [46].

The main intervention tool will be the group support sessions. To date, while some studies have assessed individual counseling for reduction of child exposure [4], none has assessed group counseling for this purpose. Thus, our consideration of group counseling represents a novel contribution. Studies in the area of smoking cessation have shown that group support, a relative newcomer to the field of tobacco control, is an effective intervention [47]. These results support the use of group therapy in the context of reducing child TSE. We will focus on the importance of protecting young children from TSE, and on ways to implement and maintain smoke-free environments for the child. We will seek to "denormalize" smoking around children, and to promote the goal of keeping homes and cars completely smoke-free. The sessions will be run by counselors experienced in working with parents of young children.

Using new communication technologies (internet forums, chat rooms, mobile phone text messages, etc.) is a relatively recent but promising concept in health promotion [48]. A review of internet-based smoking cessation programs showed that some internet-based interventions can aid smoking cessation [49]. In our program, we plan to augment the group support with a website which will provide a forum for discussion among parents, as well as a means for project personnel to communicate with parents. The idea is to use an "online/offline" approach to create a community of parents who are trying together to protect their children.

Personalized feedback in the form of biochemical test results has been previously used to decrease TSE. The Emmons study [50], one of the few studies with proven benefit, included biofeedback on the amount of nicotine present in homes. We plan to provide parents with results of biochemical measures of their child's level of TSE (specifically, hair nicotine levels) to help them internalize their children's true exposure levels.

The above components will be supplemented with a video simulation game (called "Dr. Cruz") and informational materials provided to the participants' physicians (including CEASE materials). This particular simulation proved feasible to deploy in medical waiting rooms for low-income populations in the US (California Central Valley and Kentucky), and it showed initial positive impact in terms of increased awareness of SHS in cars and intentions to change behavior [45].

Parents will be encouraged to quit smoking, but, at the same time, they will be informed that they do not have to quit to participate in the program. Parents interested in quitting will be provided with information on existing cessation services within their HMO. In addition, we may provide nicotine replacement therapy for use in promoting smoke-free homes and cars. This approach has been taken by previous researchers, with promising results [51].

### Outcome Measures

#### List of Outcome Measures

The primary outcome measure of the Phase III trial will be child TSE as assessed by hair nicotine.

Secondary outcome measures are:

1. Child TSE, as assessed by parental report

2. Adoption of voluntary smoking bans in homes and cars

3. Risk perception, attitudes and knowledge regarding child TSE

4. All child respiratory symptoms, including sneezing, wheezing, coughing, sore throat, and ear infections, as reported by the parents.

5. Parental smoking cessation, number of parental quit attempts, use of cessation medications, attendance at behavioral cessation sessions

#### Measurement

##### Measurement of TSE

• Hair nicotine levels. Hair will be cut at the scalp prior to and 3-6 months after the intervention. Several centimeters of several hairs will be taken from hair at the scalp. The proximal 1 cm of hair represents exposure during the previous month.

• Parental report of child TSE, obtained through questionnaires, including information on frequency of regular exposure, smoking habits of both parents, smoking bans in house and car, smoking behavior of frequent and occasional visitors; exposure to regular smoking elsewhere (such as in daycare or at grandparent).

##### Justification of choice of TSE measures

The measurement problem is a difficult one, and deficiencies in measurement, whether due to poor sensitivity or bias, may be a contributor to the mediocre results often observed in previous programs. If the measures used are not sensitive enough to detect small differences, then the "noise" in the measurements will obscure being able to document true effects of an intervention. If the measures are biased, and that bias is affected by intervention group (for example, with parental report), then spurious effects may be observed.

There are two approaches to measurement of child TSE: parental report and biochemical measurement. Neither method is perfect: parental report may be compromised by lack of knowledge of child exposure, lack of awareness of that exposure, or social desirability bias. The most widely accepted measure of parentally-reported exposure is number of cigarettes to which the child is exposed. In our early piloting of questionnaires, this measure proved difficult for parents to answer.

Several past studies found parental report to be sufficient [52-54], while other studies showed parents underreported exposure [55] or that such reports were unreliable [56]. In 2006, the US Surgeon General recommended use of biomarkers, particularly cotinine for assessment of SHS exposure [2]. The recent Cochrane 2006 review of programs to reduce children's exposure to SHS [4] stated "We take biological verification of exposure to or absorption of ETS [Environmental Tobacco Smoke] as the "gold standard", and recommended the use of hair nicotine for estimation of long-term exposure.

One review examined reported correlations between biochemical measures and parental report from a number of studies, and found that correlations between parental report and nicotine ranged from .22 - .75, while correlations between parental report and cotinine ranged from .28 - .71 [52] While this may be sufficiently accurate to differentiate between children of smokers and non smokers, it may not be accurate enough to detect differences in levels of exposure obtainable by intervention programs.

Our decision to use hair samples was based primarily on the higher precision of hair nicotine found in the literature [57]. Logistical considerations were also important, as the samples need to be shipped internationally for analysis, and transport delays of biologically perishable samples could result in loss of data. Indeed, a report of a very recent study includes measurement of exposure using hair nicotine [51], perhaps for similar reasons to ours.

### Statistical Considerations

#### Statistical analysis

The Phase I and II results will be analyzed primarily through descriptive methods. In the Phase III trial, the effectiveness of the intervention on hair nicotine level and parental report of child TSE will be analyzed using linear mixed models, which will take into account dependencies in the dataset due to within-clinic correlations between parents. Clinic will be included as a random effect. Baseline hair nicotine level will be included as a covariate. Potential confounding variables relating to socioeconomic status, ethnicity, and religiosity, as ascertained from the initial parental interview, will be included as covariates.

#### Sample size calculation for the Phase III trial

The final sample size calculations for the Phase III trial will be based on results from the Phase II pilot study. Preliminary calculations have been made on the basis of published data for hair cotinine, and hair nicotine [58,59]. These calculations are based on a power of 0.8 and a 2-sided alpha-level of 0.05. The published data we have used are population or sample level data on levels of hair cotinine in children exposed or not exposed to SHS, or on levels of hair nicotine in children whose parents smoke in the house, smoke only outside the house, or do not smoke at all. We made the following assumptions:

a. The aspirational goal is elimination of TSE among the children. However, our more realistic, operative goal, on which sample size calculations were performed, is a *reduction *in exposure. We define a detectable change of substantive importance as one which produces a change in exposure equivalent to one third the difference in measured hair values between children exposed and unexposed to tobacco smoke.

b. There will be some leakage of the intervention to the control group, as a direct result of the recruitment process. This is consistent with the improvements seen in twelve out of eighteen trials in the control groups, of studies included for review in the Cochrane systematic review [4]. We assume that this contamination will occur in 10% of the recruited children in the control group. This number is based on observed changes in a previous health intervention trial which was run by two of the authors of the present study [39]. Thus, the expected outcome in the control group is a weighted average of the values for exposed children (90%) and the expected change as a result of the intervention (10%).

c. An Inflation Factor (IF) is necessary to compute sample sizes due to the possibility that some of the variance is due to similarities within children attending the same clinic. The inflation factor we used is 1.145.

d. We further assume that 20% of the children recruited will be lost to follow-up.

Based on our tentative calculations, a total of eighteen clinics will be randomized equally to intervention and control groups. Thirty children will be recruited from each clinic. Five hundred and forty infants will be recruited to the study.

We assume that treatment is allocated at the clinic level, with the same number of clinics in each of the two arms. The sample size calculations are based on (1) published descriptive statistics for hair nicotine and hair cotinine, (2) specified effect sizes, (3) a projected average clinic size of 30 infants, and (4) a projected response rate of 80%. The sample sizes are also adjusted using an inflation factor to account for the correlation between children attending the same clinic.

##### Sample size formula

The total sample size is calculated by the following standard formula: [60]

Here z_a _= 1.96 and z_b _= 1.28, for 90% power at the two-sided 0.05 level, Δ is the relevant effect size, and σ is the relevant standard deviation (SD). The sample size is then multiplied by the Inflation Factor (IF) and divided by the expected response rate of 0.8.

##### Specification of the effect size

The effect of SHS can be expressed as the ratio of the hair nicotine or cotinine level between exposed and unexposed populations. We assume that the program will reduce this ratio by 25%, 33%, or 50%. We further assume that, due to leakage of the program to the control group, the ratio will be reduced in the control group by an amount equal to 10% of the reduction in the treated group (i.e. 2.5%, 3.3%, or 5%).

We perform the calculations on a logarithmic scale. Denote by R the reduction achieved by the program. The effect size (i.e. difference between treatment groups) on the logarithmic scale is then given by

For R = 0.25, 0.33, and 0.50, the corresponding Δ values are Δ = 0.26, 0.37, and 0.64.

##### Estimation of the Inflation Factor (IF)

The IF is determined by the intraclass correlation coefficient (ICC) and the number of children per clinic [61]. The formula is IF = (1+(m-1)ρ), where m is the average number of children per clinic and ρ is the ICC. As was done by the authors of a previous study, we assume ρ = 0.005. Our assumed clinic size is m = 30. Hence the IF is given by IF = 1+29*0.005 = 1.145.

##### Estimation of the standard deviation (SD)

###### Hair Nicotine

Our endpoint is log hair nicotine level. Al-Deleimy et al. [59] presented geometric means and associated confidence intervals for hair nicotine. These results were generated by computing arithmetic means and associated confidence intervals for log hair nicotine and then backtransforming to the original hair nicotine scale. From the results presented in the Al-Deleimy paper, we can derive the SD for log hair nicotine in the Al-Deleimy study.

Al-Deleimy et al. presented data for nonsmoking families, parents who smoked only outside the house, and parents who smoked inside the house as well as outside. Table 2 presents the published results and the corresponding SD's for log hair nicotine. Given the above results, we estimate the SD for log hair nicotine at σ = 1.00.

**Table 2:** Hair Nicotine Results From Al-Deleimy et al

	Published N	Published Geometric Mean	Published Confidence Interval	SD for Log Hair Nicotine
No exposure	101	0.58	[0.49,0.68]	0.84
Smoke outside the house	69	2.63	[2.03,3.40]	1.09
Smoke inside the house	127	5.62	[4.60,6.86]	1.15

###### Hair Cotinine

Florescu et al. [58] presented descriptive statistics for hair cotinine for various populations. We focus on the population of children, either exposed or unexposed to tobacco smoke.

Florescu et al. state on page 440 of their paper that hair cotinine did not follow a normal distribution, and they therefore presented geometric means and some other statistics of interest. We take log hair cotinine as our endpoint, and assume that it is normally distributed. From standard mathematical results for the lognormal distribution, we can use the results in the Florescu et al paper to calculate estimates of the mean and SD of log hair cotinine.

The estimated means for log hair cotinine are in close agreement with the reported geometric means for hair cotinine previously reported [exp(-1.59) = 0.20 vs. 0.19, exp(-0.84) = 0.42 vs. 0.48]. Based on the results, we estimate the SD for log hair cotinine at σ = 1.15.

##### Calculated Sample Size

Given the foregoing development, the calculated total sample sizes for log hair nicotine and log hair cotinine at the various levels of R are as follows (rounded up to the nearest 10) as seen in Table 3.

**Table 3:** Calculated Total Sample Sizes

	Log Hair Nicotine	Log Hair Cotinine
R = 0.25	880	1,160

R = 0.33	450	600

R = 0.50	150	200

The calculated sample sizes are higher for log hair cotinine than for log hair nicotine. This is consistent with the claims that hair nicotine is more sensitive than hair cotinine.

Using R = 0.33 and taking log hair nicotine as the endpoint, we get a total sample size of 450 infants. Since the number of infants has to be an even multiple of the clinic size, which we are assuming is 30, we get a total sample size of 480. We increase this to 540 infants as a security measure against possible clinic dropout and the uncertainty in the estimates used. We thus set the tentative total sample size at 540 infants (9 clinics in each of the two arms, with 30 infants per clinic). The final choice of endpoint and sample size will be made on the basis of locally-collected data.

### Ethical approval and trial registration

Institutional Review Board approval has been obtained from the Ethics Committee of the Sackler Medical Faculty at Tel Aviv University. All participants will provide written informed consent. The trial is registered with the NIH trials registry database (ClinicalTrials.gov Identifier: NCT01335178).

## Discussion

This paper describes the planning process, a proposed program and its theoretical rationale, and an evaluation design of an innovative intervention to protect young children from tobacco smoke exposure. The primary challenges of this work are to (1) develop an effective theory-based intervention, and (2) to measure the effects with sufficient accuracy and precision to detect a true interventional effect.

Existing reviews of published reports show, overall, that the effects of similar interventions were either small or nonexistent [13,62-65]. This is consistent with many findings of health promoting community based interventions [66]. Findings could be due to one or a combination of three reasons: either the intervention programs tested were ineffective, the programs were effective but were not sufficiently well implemented, or the measurement of the results was not sufficiently accurate to detect the effects of the intervention.

Our developed program builds on recent ideas of others from the fields of parenting, health communication (new media and online/offline approach), smoking cessation (group therapy), and reduction of exposure (CEASE materials for involvement of physicians, biochemical feedback for internalization about personal exposure levels, Dr. Cruz for education about TSE in cars with smokers, and possible use of NRT for maintaining smoke-free homes and cars). We avoid the common failure of using "one shot" or very short-term interventions, by using multiple group meetings augmented by a website. Our developmental plan ensures that the full trial will not go forward until we have evidence of feasibility and some indication that it is effective (albeit in an uncontrolled manner).

## Conclusions

In this article we present our rationale for and method of program development, proposed intervention plan, and evaluation plan. We describe a design that draws on social marketing and behavior-change approaches that is parent-focused and allows parents to join the program, even if they initially do not intend to quit smoking, and offers them a group support system that will address their concerns and needs. We identify important challenges faced by researchers interested in developing and evaluating interventions to reduce child exposure to tobacco smoke face, and offer specific strategies to address them. The proposed approach and its rationale can serve to contribute to building the science base for the development and testing of intervention approaches for health-promoting practices, and in particular the protection of children from tobacco smoke exposure.

## Abbreviations

ETS: Environmental Tobacco Smoke; HMO: Health Maintenance Organizations; ICC: Interclass correlation coefficient; IF: Inflation Factor; NIH: (United States) National Institute of Health; NRT: nicotine replacement therapy; SD: standard deviation; SHS: Secondhand smoke; SHSe: Secondhand smoke exposure; SMS: short message service; TSE: tobacco smoke exposure

## Competing interests

The authors declare that they have no competing interests.

## Authors' contributions

LR initiated the study, designed the study with DZ, NG, and MH, wrote the initial draft of the manuscript, and bears overall responsibility for the project. NG, MH, VS, JW, NK, and MBN made important contributions to the development of the intervention (Of particular note: NG contributed the idea of a social marketing approach, VS conceived of and wrote the initial version of the project website, JW contributed materials for use with pediatricians, NK contributed use of the simulation game "Dr. Cruz", and MBN reviewed the literature). ST, JR, and UR contributed to program and evaluation design in a real-world context. DZ performed the sample size calculations and contributed to the statistical analysis plan. MBN and VS contributed to questionnaire development and CV wrote the hair sampling protocol. DZ, NG, and MH contributed to the writing of the manuscript. All authors critically revised the manuscript and approved the final version.

## Pre-publication history

The pre-publication history for this paper can be accessed here:

http://www.biomedcentral.com/1471-2458/11/508/prepub
